# Supplementary material for: Droplet-based microfluidic platform for high-throughput screening of Streptomyces
Source: Commun Biol. 2021 May 31;4:647. doi: 10.1038/s42003-021-02186-y (PMC8166820; doi:10.1038/s42003-021-02186-y)
Supplement: Supplementary file 2 — Supplementary Information [file 42003_2021_2186_MOESM2_ESM.pdf]

Supplementary information

Droplet-based microfluidic platform for high-throughput screening of *Streptomyces*

Ran Tu[1,2,#], Yue Zhang[1,2,#], Erbing Hua[3], Likuan Bai[2,3], Huamei Huang[2,3], Kaiyue Yun[2,3], Meng Wang[1,2,*]

***Corresponding author:**

Dr. Meng Wang, E-mail: wangmeng@tib.cas.cn

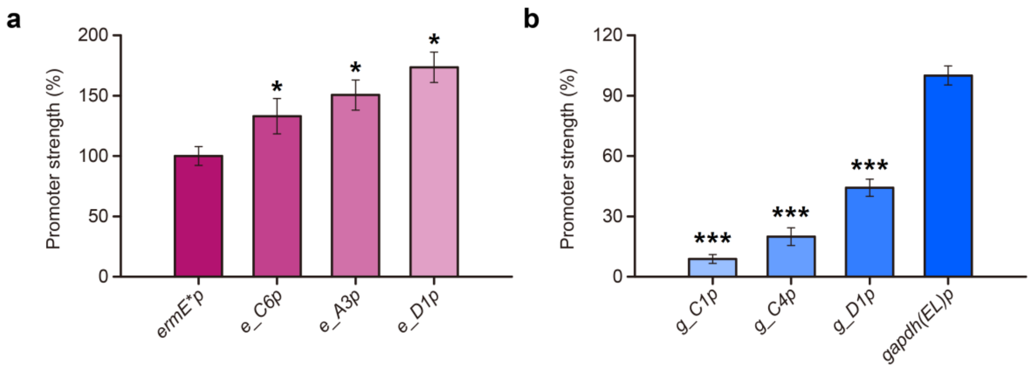

Supplementary Figure 1. Relative strength of promoters from e-lib (a) and g-lib (b) in 24-well plate. Wild-type *ermE**p* and *gapdh(EL)p* were normalized to 100% for e-lib and g-lib variants, respectively. For each experiment, three replicate samples were analyzed (n=3). Error bars represented the standard deviation. *$p \leq 0.05$ and ***$p \leq 0.001$ (Student's two-tailed t-test). Source data were provided in Supplementary Data 1.

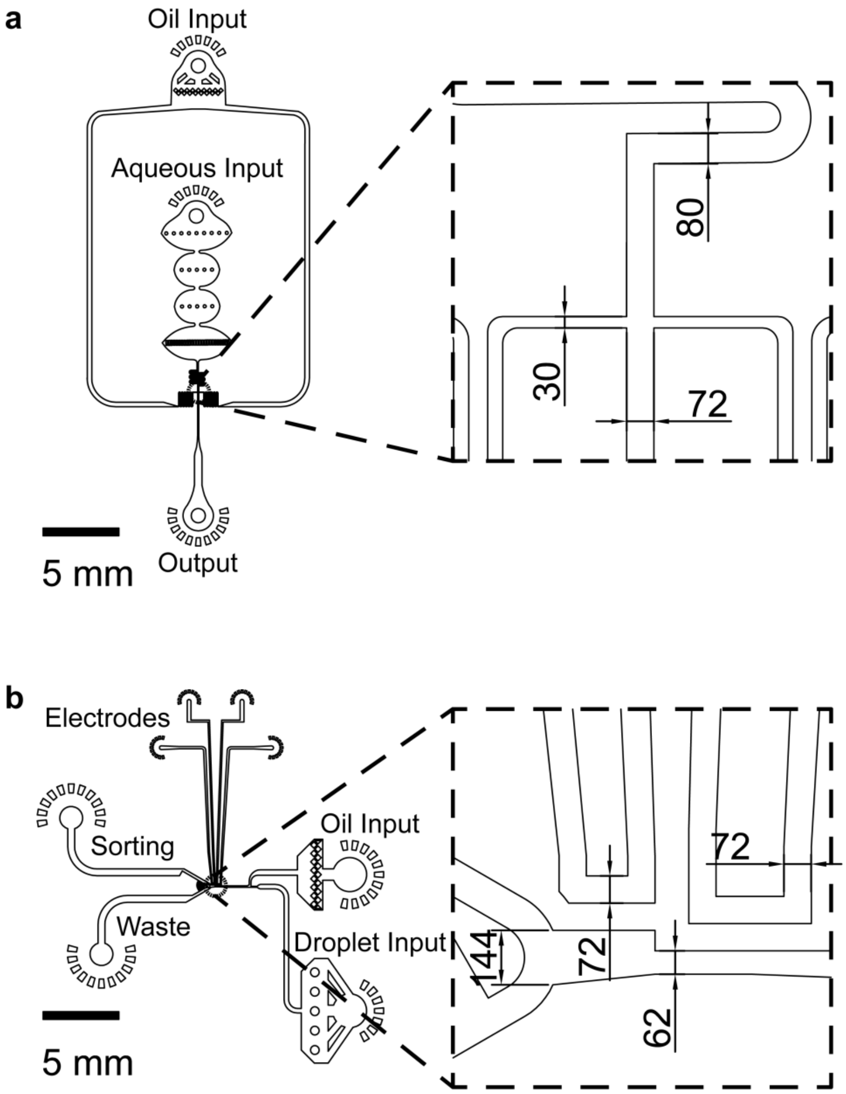

Supplementary Figure 2. Schematics of the droplet making device (a) and the droplet sorting device (b). Channels are 50 μm deep. The specific size of the junction was marked in microns (μm).

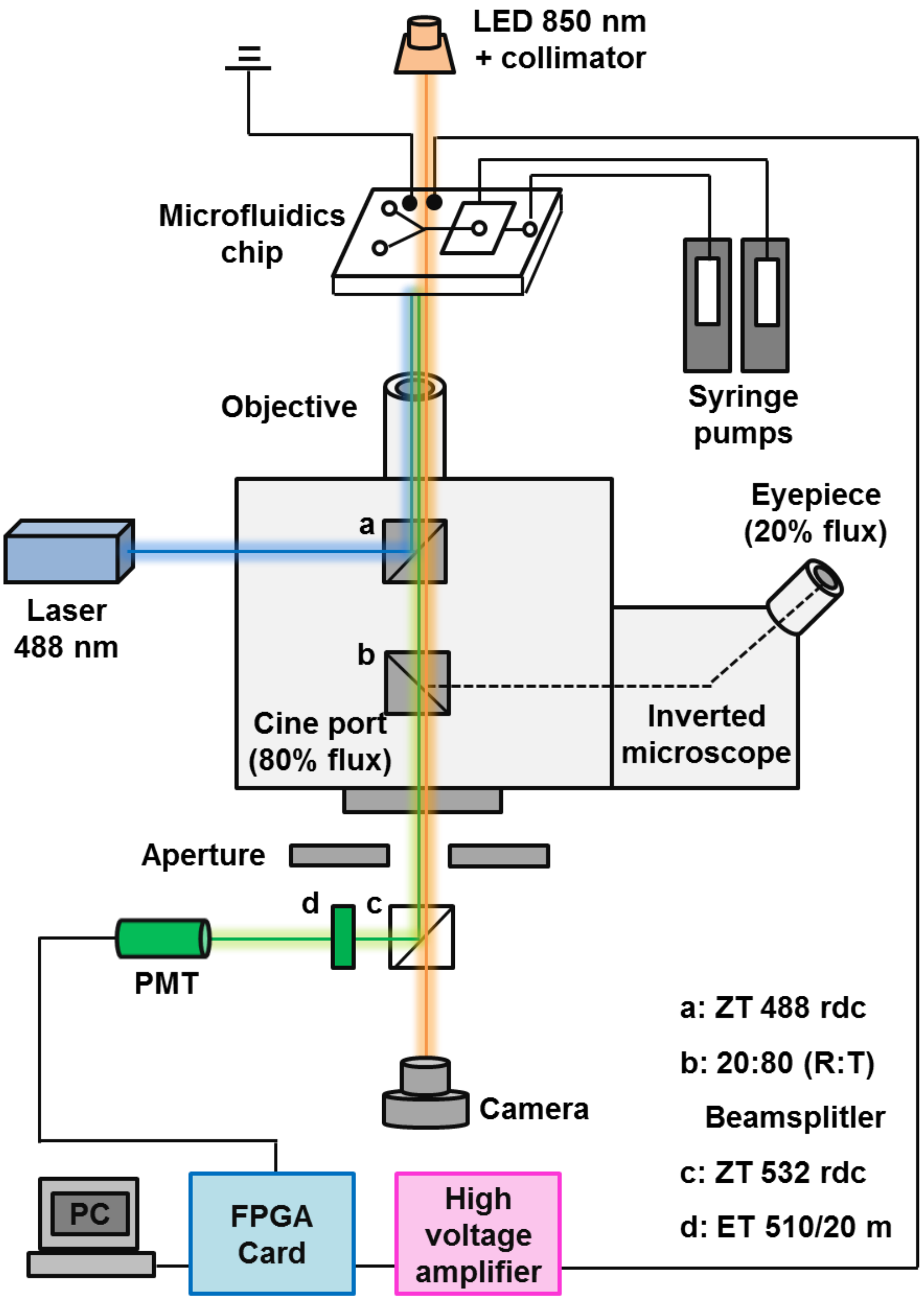

Supplementary Figure 3. Schematic of the optical setup of microfluidic system. The orange line indicated the 850-nm light used for observation by the camera. Microfluidic chip was exposed under 488-nm laser (blue line). The 520-nm fluorescent light (green line) emitted by droplets was detected by the photomultiplier tube (PMT). The data was acquired by the FPGA data acquisition card (blue box) to record the fluorescence intensity of droplets and analyzed by the computer (PC). High voltage amplifier (red box) was used to apply a voltage to the droplets for deflection and sorting.

Supplementary Table 1. The sequences of promoter variants sorted from e-lib and g-lib. -35 regions were indicated by solid lines, and -10 regions were indicated by dot lines.

Promoter	Sequence (5'-3')	Fold-change	Relative strength
*ermE*p*	TTGACGGCTGGCGAGAGGTGCGGGGAGGAT	100%	3.4
e_C6p	TTGACGCATTTGCAGCGCTTTGGGGAGGAT	133.5%	4.5
e_A3p	TTGACGTACTGTCTGTCGATGGGAGAGGAT	343.2%	11.6
e_D1p	TTGACGAAAGAGTGGGATGTGGGGGAGGAT	347.9%	11.8
g_C1p	TTGCAGCACACACTCGGAACGTCATATGAT	10.1%	10.1
g_C4p	TTGCAGAAAGAGGAAGCAAGAAAATATGAT	24.7%	24.7
g_D1p	TTGCAGCTACATTGGATTATCTGGTATGAT	94.6%	94.6
gapdh(EL)p	TTGCAGCATCTGGGCGGCTACCGCTATGAT	100%	100
