# Supplementary material for: Droplet-based microfluidic platform for high-throughput screening of Streptomyces
Source: Commun Biol. 2021 May 31;4:647. doi: 10.1038/s42003-021-02186-y (PMC8166820; doi:10.1038/s42003-021-02186-y)
Supplement: Supplementary file 3 — Description of Additional Supplementary Files [file 42003_2021_2186_MOESM3_ESM.pdf]

Description of Additional Supplementary Files

File Name: Supplementary Data 1
Description: Source Data

File Name: Supplementary Data 2
Description: Sequences of the native and heterologous promoters used in this study.

File Name: Supplementary Data 3
Description: Strains and plasmids used in this study.

File Name: Supplementary Data 4
Description: Primers used for vector construction.
